# Long-distance social relationships can both undercut and promote local natural resource management

**DOI:** 10.1098/rstb.2022.0269

**Published:** 2024-01-01

**Authors:** Anne C. Pisor, Monique Borgerhoff Mulder, Kristopher M. Smith

**Affiliations:** ^1^ Department of Anthropology, Washington State University, Pullman, WA, USA; ^2^ Department of Human Behavior, Ecology, and Culture, Max Planck Institute for Evolutionary Anthropology, 04103, Leipzig, Germany; ^3^ Santa Fe Institute, New Mexico, 87506, NM, USA; ^4^ Department of Anthropology, University of California at Davis, Davis, 95616, CA, USA

**Keywords:** cooperation, collective action, social capital, natural resource management, human evolution

## Abstract

The management of large common-pool resources, like fisheries and forests, is more difficult when more people and more communities can access them—a particular problem given increased population sizes, higher mobility and globalized trade in the Anthropocene. Social relationships spanning communities, such as kin relationships, business or trade relationships and friendships, can make management even more challenging by facilitating and transmitting norms of overharvesting. However, these long-distance relationships can also bolster management by transmitting norms for sustainability, promoting interdependence and laying the groundwork for nested management systems. Here, we review the negative and positive impacts of long-distance relationships on local natural resource management (NRM), providing illustrative examples from our field research on forest and fisheries management in Tanzania. Drawing on the evolutionary literature, the development literature and our field data, we offer suggestions for how development partners can avoid the pitfalls of long-distance relationships and how they can use or even deliberately foster long-distance relationships to promote successful local NRM.

This article is part of the theme issue ‘Evolution and sustainability: gathering the strands for an Anthropocene synthesis’.

## 1. Introduction

Given ongoing challenges posed by the Anthropocene, natural resource management (NRM) is one of the pressing issues of our time. At the centre of the crisis are common-pool resources (CPRs), natural resources like forests, fisheries and watersheds that can be depleted and from which it is difficult to exclude users [1]—especially when the CPR can be accessed by multiple communities [2]. Each of these resources poses a ‘sustainability frontier’ requiring social mechanisms of environmental control [3]. To prevent depletion in both the Global North and Global South, development partners (e.g. governmental or non-governmental organizations (NGOs)) often use design principles such as clearly identifying users who help create fair rules with ‘costs proportionate to benefits' for resource extraction [4]; these rules should reflect local conditions and be designed and enforced by users themselves [2,4]. Communities (socially defined groups, typically of spatially clustered dwellings like neighbourhoods) often provide easy demarcation of users and resource boundaries for identifying common-pool resource units (CPRUs). Communities also often have existing institutions, or rules and norms [5] that directly address or can be repurposed for community-based NRM. Linking CPRUs to communities has proven to be a highly successful strategy for steering CPR management towards success [6].

However, collective action is more challenging when more communities and more people can access a CPR. Major breakthroughs in institutional economics [7,8] and some areas of anthropology (e.g. [9]) led conservation and development partners to valorize the community as an isolated locus of action [10], according to the idea that decentralizing to the community level would solve many problems. As the challenges of the Anthropocene have escalated, conservation scientists (concerned with biodiversity) and development agencies (tackling broader issues of sustainability) have thus increasingly fixed their gaze on community action [11]. However, this local turn also promotes a tendency to ignore connections between resources and between the people who use them [12], connections which are only becoming more common in the Anthropocene. When community-based cooperation fails, it creates externalities that can undermine multi-level collective action with global-scale implications [3,13–15].

As recognized by many scholars studying complex, cross-boundary and large-scale resource systems such as river basins and delta areas [16], there is an increased cost of monitoring more people, possibility of inconsistency in enforcement and differences across communities in goals for NRM, usage patterns and norms [2]. One solution supported by both theoretical models and empirical work [17–19] are polycentric arrangements, or ‘small units nested in larger systems’ [2], where the small units are often CPRUs or a combination of CPRUs and government entities that can facilitate coordination [17]. For example, water catchments (e.g. in southeast Queensland, Australia) often have interdependent CPRUs—different aquifers, which may rely more or less on groundwater versus surface water [20]. Nested, polycentric structures built around CPRUs reduce the costs of monitoring, facilitate punishment, coordinate goals across communities and ensure that management rules and the way they are enforced match the ecology and institutions of each community [18,19,21,22].

Here, we discuss the relevance of an evolutionary perspective to the success of polycentric NRM when multiple communities are involved, focusing especially on what we call ‘long-distance relationships'—social ties that cross community boundaries, sometimes even crossing continents—that can impact the movement of capital and cultural information with implications for individual well-being. We review the significance of long-distance relationships in ancient and contemporary populations across the globe, and how these relationships can foster fitness interdependence that can hinder or help larger-scale collective action. We bring in perspectives from the development literature and from our ethnographic experiences with communities in Tanzania, providing illustrations of how long-distance relationships can both bolster and undercut successful local NRM in polycentric institutions. Study of these long-distance relationships in the Global South has import for the Global North as well, where, in the context of water resource management for example, long-distance relationships can transmit successful management institutions [23] and serve as the backbone for polycentric systems of management, even in urban spaces [20]. In short, taking the long view of human history, we ask what an evolutionary perspective can tell us about when long-distance relationships promote or undercut NRM, and how this can inform institutional design in the Anthropocene, whether in the Global North or the Global South.

## 2. Fitness interdependence and long-distance relationships

Polycentric arrangements can be more successful when built on top of existing connections that entail fitness interdependence. Fitness interdependence refers to shared outcomes between two or more entities because of mutual influence on each other's success, which can be generated in many ways, including via kinship, reciprocity [24] and institutions [25,26]. For example, civic organizations can create fitness interdependence, as can marriages (e.g. between in-laws) and business partnerships. Entities with fitness interdependence can be individuals or groups, like communities or CPRUs, where success can refer to group persistence, expanded group membership or the spread of socially transmitted traits—like the design of polycentric institutions or aspirations for sustainable management—from one group to another [27,28]. Fitness interdependence is a unifying principle for explanations of cooperation [25], providing a common framework to understand how different mechanisms that tie entities' outcomes to one another can encourage cooperation.

Often, fitness interdependence is cultivated through shared risk management. To manage risk, people maintain social networks with nested structure, in which the well-connected core around an individual helps them manage risks that are more idiosyncratic across households, while their sparsely connected periphery, involving connections that are often long-distance, helps them manage more-correlated risks [29,30]. These long-distance relationships frequently span community boundaries [31,32] and thus can create fitness interdependence across communities. Indeed, some of the most important risk-management networks in the twenty-first century are remittance networks, with resources often flowing across country borders and even continents [33]. These risk-management relationships can include phenomena like trans-local ties (e.g. locality to locality connections across borders [27]), long ties (which bridge social networks [34]) and the ties maintained by individuals high on cosmopolitanism or global consciousness, or with superordinate or global identities [35–38].

Long-distance relationships have probably been part of human life since at least 300 000 years ago, if not earlier, helping humans manage the climate variability that characterized the Late Pleistocene (e.g. [39]) by enabling people to access resources at a distance [31,32,34,40] and to reduce the impacts of local resource shortfalls through strategies like trade, gifts and visitation [31,41–43]. For past and present populations engaged in subsistence production, long-distance relationships provide access to resources key to health and food production like salt, stone and medicines [44]. Trade, for example, enabled the regular movement of obsidian over 100 km in East Africa 125 000 years ago [32]. Likewise, long-distance relationships are often central to populations participating in market economies, both historically (e.g. as facilitated by merchant guilds [31]) and today (e.g. trans-local divisions of labour across countries [33]). Furthermore colonial expansion, from prehistoric periods to the present, plays a role in both building and undermining long-distance relationships: settler colonialists forged long-distance relationships between nodes in colonial empires, and indigenous long-distance relationships were undercut by subjugation and forced displacement [45].

In the Anthropocene, long-distance relationships can provide access to jobs [46], remittances of money [38], business loans [47] and business connections [19]. Long-distance relationships often help with recovery from contemporary shocks, like tsunamis and the collapse of fisheries [48,49]. They can be formed and maintained even more easily than before—if not through air or ground transportation, then through social media [36,46], where ties between people with fewer shared contacts can be among the strongest [34]. Perhaps unsurprisingly, given increased mobility and coordination via phone and internet, long-distance relationships can deplete natural resources [50], including fisheries [51] and forests [52].

**Figure 1.**
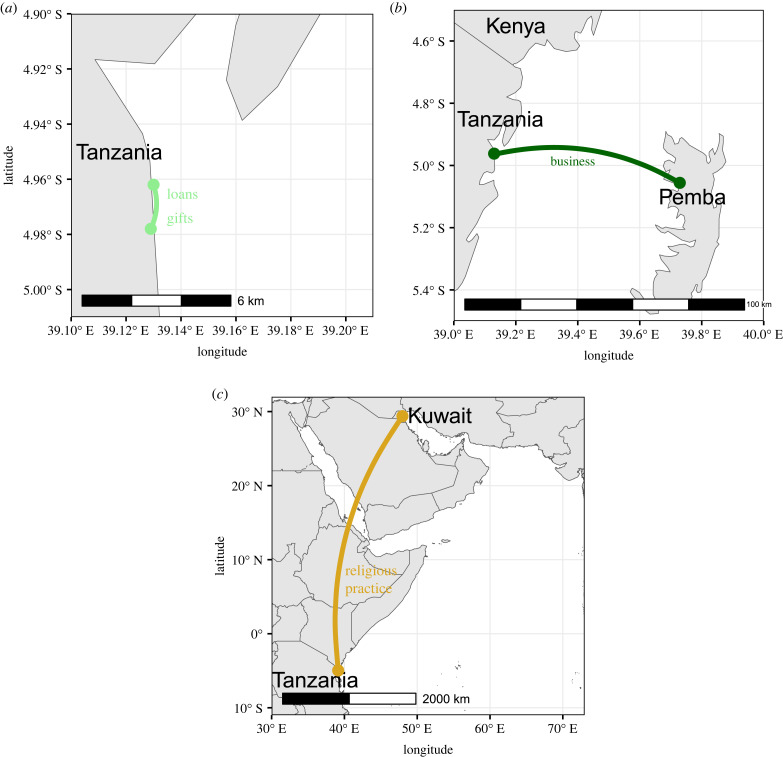
Examples of long-distance relationships, and the things they transmit, between individuals in Tanga, Tanzania and alters living elsewhere, including in the next community (*a*), in Pemba, Zanzibar (*b*), and in Kuwait (*c*). Credit for map rasters: Natural Earth (https://www.naturalearthdata.com/). (Online version in colour.)

Because long-distance relationships are all about risk management, the geographical scale of risk an individual has to manage, and thus the distance a long-distance relationship must span, will vary by the resource in question and a variety of constraints [50], including the availability of technology, the cost of travel and border crossings, laws, ethnic tensions and more [31] (figure 1). Long-distance relationships can cross the boundaries of ethnic or religious groups, regions or countries, but are often easier to form with same-group members because shared norms can reduce transaction costs [31].

Long-distance relationships take many forms—for example, they can exist between siblings or cousins, friends, godparents, business partners and even members of government institutions—but each of these long-distance relationship types often entail fitness interdependence. Long-distance relationships are commonly long term, as the sharing they involve, and the investment it takes to maintain them (e.g. gifts, visits), may only result in net benefits over long periods of time. They can be costly: people may not have the time or resources to form them even if they want to [50], and institutions themselves can prohibit them [53]. However, when long-distance relationships are important to risk management, individuals may be more interested in forming them [38,54], and group-level institutions often evolve to support them [44,50]—for example, through norms that lower the cost of long-distance relationships by reducing transaction costs [53,54] or by brokering long-distance relationships that benefit all community members, e.g. through middlemen [55,56].

For a long-distance connection (alter) to provide net benefits to an individual, two things matter. First, the alter must be able to provide when something is needed, so the alter must persist (e.g. if the alter is a person, they must be available; if a group or a community, it must not dissolve) and retain resource access and productivity [57,58]. Second, the *resources* the individual may need must also persist. For these reasons, long-distance connections have a vested interest in each others’ resource access and outcomes, and thus each individual is motivated to attend to how their actions affect their alter; this may contribute to the higher levels of global prosociality often measured in research on cosmopolitanism, superordinate identities and global consciousness [36,38]. However, the degree to which this vested interest affects decision-making is contingent on time horizons and visibility. First, when need is acute, long-term outcomes for alters and their resources may weigh less heavily in an individual's decision-making [59]. Indeed, what some call the ‘short-termism trap’ is a common culprit in undermining NRM generally [60]. Second, if the negative impacts of individual behaviour occur at a distance and are thus more difficult to monitor, they may feed back into individual decision-making less than more localized impacts [60]. The variability in the effect of fitness interdependence on decision-making is why long-distance relationships can have both negative and positive impacts on cooperation beyond the dyad, such as on the cooperation required for local NRM.

## 3. The negative and positive impacts of long-distance relationships on natural resource management

**Figure 2.**
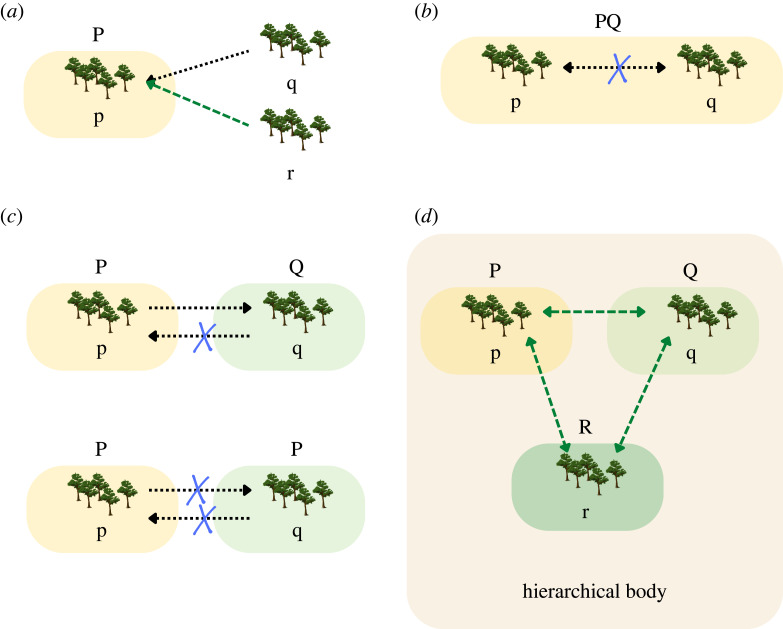
Four examples of the impacts of long-distance relationships on natural resource management. Stands of trees each represent a CPRU and are labelled as p,q,r; all such units are assumed to be asynchronous along some dimension (e.g. resource availability and species present). Shaded rectangles denote the management institutions P,Q,R. Lines denote the impact of long-distance relationships on a given CPRU (dots with negative consequences, dashes with positive consequences) and x's denote the interruption of these impacts. (*a*) Users in q and r may support (r) or undercut (q) the CPRU p with institution P. (*b*) To stop users in p harvesting from q, and vice versa, users of p and q form a joint management committee (PQ). (*c*) Institution P is effective at stopping leakage, but Q is less effective; users of q may adopt institution P to reduce leakage. (*d*) An umbrella organization coordinates the activities of the management institutions (P,Q,R) of three CPRU (p,q,r). Credit for images: Canva. (Online version in colour.)

Reviewing the development and evolutionary literatures elucidates how long-distance relationships, while often generating benefits for the individuals connected, can have both negative and positive impacts on the management of large CPRs like forests and fisheries (figure 2). To provide just a few examples: first, when individuals in long-distance relationships help one another, resources may flow away from CPRUs, often moving from well-resourced communities to more impoverished ones [46], which can exacerbate natural resource depletion [61]. Long-distance relationships can thus undercut or enhance protection activities conducted by local management institutions—e.g. institution P in figure 2*a*. When they undercut protections, standard commons management theory would attribute this to poor social boundaries [4]. Second, when users from one CPRU are harvesting from an alter's CPRU and vice versa, the two CPRUs may voluntarily form a joint management committee to reduce harvesting (e.g. p and q voluntarily form PQ; figure 2*b*). Third, individuals may harvest resources in their alter's CPRU to evade regulations on harvests in their home communities—a phenomenon called leakage (e.g. users of p harvesting from q; figure 2*c*) [47]. If leakage occurs at high enough levels, it can cause NRM institutions in affected communities to fail [62,63]; the community experiencing increased harvests may then adopt the regulations of their alter to curtail leakage (e.g. users of q adopt institution P). Fourth, long-distance relationships can help foster collective action between CPRUs by instigating cooperation or forging additional connections between entities in a polycentric system [48,64], coordinating activities of management institutions across multiple CPRUs (e.g. of institutions P,Q,R for CPRUs p,q,r; figure 2*d*) and thus reducing leakage and reducing transaction costs. Long-distance relationships can foster horizontal connections, as between CPRUs in this example [49] or vertical connections between villagers and district governments in the case of co-management [51,52].

In short, long-distance relationships can prove both an asset and a nuisance, or worse, to organizations hoping to foster successful local NRM. To provide real-world examples of how long-distance relationships can bolster or undercut successful NRM, we turn to two examples of polycentric management from the development literature: Community Forestry Management Agreements and Locally Managed Marine Areas.

## 4. Long-distance relationships and polycentric natural resource management in Tanzania

Long-distance relationships are a defining feature of Swahili culture in East Africa, which developed through centuries of exploration, trading and diplomacy with different maritime communities across the Western Indian Ocean—first, with Persia, Arabia, Africa and the Indian subcontinent, and later including Europe [65–67]. Marriage and business ties solidified these relationships, which over the years facilitated the movement of diverse goods, including salt, gold, slaves, ebony, ivory and cloves [68,69]. These long-distance relationships transformed natural resource extraction in the Western Indian Ocean, a process impacted by multiple colonizing powers [66]. For example, on the Zanzibari island of Unguja, increases in clove production in the nineteenth century meant agricultural and foraging options dwindled, increasing demands for fish and mangrove poles as fishing and pole cutting became specializations [70]. More generally, long-distance relationships have shaped patterns of resource use and access across the Western Indian Ocean before and during the Anthropocene [67].

Against this background, we use two examples drawn from our own fieldwork on CPRs in this region—specifically forest-dependent communities on the Zanzibari archipelago island of Pemba and fishing communities in Tanzania's northern Tanga region—to examine how long-distance relationships can both bolster and undercut successful community NRM. These insights come from our ethnographic experience in both places, including observations and formal and informal interviews. (For further details on these contexts and our research methodologies see [54,71].) While we draw on local examples from our fieldwork in Tanzania, these local examples should be seen in their global context. For example, the forests of Zanzibar were impacted by early Islamic trade and the arrival of rice; greatly diminished by the nineteenth century spice trade in cloves, intermittently both harvested and protected by British colonial interests; and most recently exposed to various foreign-led conservation initiatives [71] including carbon accreditation [52]. Similarly, fisheries in the Global South, including Tanzania, have been identified as ‘fisheries-conservation hotspots' because of their high biodiversity and growing exploitation relative to wealthier countries in the Global North [70]. In short, in the Anthropocene, global forces impact local resource management and long-distance relationships, which in turn have impacts on global outcomes; no one component can be considered in isolation.

### (a) Forest community management on Pemba

The Zanzibari archipelago is a semi-autonomous territory in political union with Tanzania, lying just off the mainland coast. It was colonized in the mid-nineteenth century by the Sultans of Oman who engaged in extensive maritime trade in dates, cloves, rice, millet, sugar and pearls, importing slaves from the African mainland and elsewhere. To this day the inhabitants of the northern island of Pemba, people of mixed Arab, Persian, Swahili and mainland ancestry, have active ties to the Arabian peninsula (*Arabuni*), to Unguja (the richer and more market-integrated of the two Zanzibari islands) and to the African mainland (*bara*) through friends and relatives who migrate for business, farming or fishing. Ties among individuals in different communities, both in adjacent zones and in other parts of Pemba, are also very strong. This reflects the small size of the island (988 km^2^), the marked heterogeneity of its closely braided ecological zones [71], the extensive fragmentation of individual land ownership (owing both to Islamic inheritance rules and land redistribution at the 1964 revolution [72]), dispersed homes (often in association with polygyny), affinal relationships and more recently adopted microloan institutions.

In this context, we review the impacts of such ties on the success of community forest management in Pemba, where we have been working since 2015. More specifically, we focus on potential dynamics among communities adopting Community Forest Management Agreement institutions as part of a carbon-trading intervention (Reducing Emissions from Deforestation and Land Degradation (REDD+) [52,73]), linked to a multi-sited, multi-level global carbon market. Under this programme, designed primarily by heavy carbon-emitting nations to slow emissions at a global scale and embraced by the autonomous Zanzibari government, local communities in Pemba were to be rewarded with cash payments proportional to the reduced emissions resulting from their efforts (‘additionality’ [74]). Here we disregard the debates regarding the use of international funding to remedy global inequities (e.g. [75]), the specific role of the state [52], and indeed the potential impact of carbon payments on ‘crowding out’ moral commitments to conservation within local communities [76]. Rather we focus on how rural Pembans with Community Forest Management Agreements established an institution—the *Shehia* Conservation Committee—to manage the collectively held forested area, CPRU, within the *shehia* boundaries, and the impact thereon of long-distance relationships.

In *shehia* with Community Forest Management Agreement institutions, community members on the committee can patrol the area, exact fines from community members and outsiders for illegal infractions, and engage in reforestation, community education and other activities designed to ensure forest protection and sustainable harvests. These Shehia Conservation Committees themselves are nested into the Jumuiya ya Uhifadhi Misitu ya Jamii Zanzibar (JUMIJAZA), a local organization that coordinates across Shehia Conservation Committees, thereby providing a polycentric governance structure.

The success of these Community Forest Management Agreements has been variable [52] and seriously jeopardized by the failure of carbon payments to materialize [73,77]. However, the potential dynamics among members of different shehia—including coordination (facilitated through JUMIJAZA), imitation and theft—are central to how community forest management institutions may thrive and spread across the island as a result of the carbon-trading intervention [78]. Accordingly, although long-distance relationships were not the focus of an explicit research strategy, our evaluation of the success of, and challenges to, Community Forest Management Agreements reveals various pathways whereby ties between different *shehia* have the potential to both strengthen and undercut local action, as outlined in figure 2.

*First, there is plenty of evidence that individuals in a community with strong Community Forest Management Agreement institutions can use their long-distance relationships to increase private harvests* (figure 2*a*). Community forestry management inevitably opens a window to free-riding: reaping the benefits of the well-managed forest without paying the cost of stinting. In many Pemban communities with Community Forest Management Agreements, individuals cooperate with outsiders to illegally harvest their own forest, and gain either directly or indirectly as a consequence. The scenarios are varied—an associate elsewhere who owns an (illegal) chainsaw, a friend in a forest-poor part of the island needing timber to extend her home, a partner in the fishing sector seeking to build a new boat or a political alliance in need of nurturing. In all such cases long-distance relationships can undercut community forest management institutions and secure private gain, and can extend beyond the purely local: business associates on Unguja may be seeking timber to construct a tourist lodge, or relatives in Oman demanding forest clearance so as to plant more clove trees.

*Second, personal links among Shehia Conservation Committee members in adjacent shehia permit coordination of their forest protection activities* (figure 2*b*). In one case, two adjacent shehia had been stealing from each other—that is, there was leakage, which is often facilitated with the approval and/or logistical support of friends and relatives who live in unprotected areas. Recognizing this leakage problem, and with the objective of minimizing damage to their own forest so as to maximize anticipated carbon payments, these two neighbouring Shehia Conservation Committees voluntarily formed a joint management committee to control leakage. Members of the two Shehia Conservation Committees used their personal long-distance relationships to coordinate their joint activities, combine patrols and effectively created an endogenous polycentric institution—a single Shehia Conservation Committee managing both forests. In this way, forest management outcomes improved in both *shehia*, even without the specific coordination of JUMIJAZA.

*Third, whether directly or indirectly, long-distance relationships can promote the adoption of Shehia Conservation Committee practices beyond the initially targeted shehia* (figure 2*c*). This can happen in two ways. First, long-distance relationships between *shehia* with different institutional arrangements can transmit norms for resource management, leading to endogenous institutional spread [15,78,79] and changes in collective decision-making as communities learn from their neighbours' experiences [80]; such learning processes can, in principle, be encouraged through an organization like JUMIJAZA. Second, where a *shehia* with a strict Shehia Conservation Committee borders a *shehia* with no (or weak) forest protection institutions, there is a strong temptation to avoid risk of punishment in the home forest and harvest in the unprotected adjacent *shehia*. Leakage can, somewhat counterintuitively, promote the first path [78]: as leaders or environmentally minded individuals in these neighbouring (encroached-on) communities recognize the impact of excessive harvesting, they may approach JUMIJAZA and/or the government for assistance in developing their own Shehia Conservation Committee institutions, including patrols, so as to better protect their forests. This dynamic accounts in part for the continued growth in the number of *shehia* with, or seeking, Community Forest Management Agreement institutions across Pemba despite the absence of carbon payments [49], whether done through the government or JUMIJAZA. Notably, however, more perverse outcomes are possible: if leakage is high, Shehia Conservation Committee institutions may fail, as we demonstrate both through agent-based modelling and empirical work [62].

*Finally, it seems likely that long-distance relationships both contribute to, and result from, the efforts of development partners to establish polycentric institutions* (figure 2*d*). In Pemba, the Shehia Conservation Committees were linked to JUMIJAZA, as well as to the government forestry department. Both bodies have contributed to coordinating NRM strategies across CPRUs, reducing leakage and facilitating depersonalized punishment, thereby lowering transaction costs [52]. Though we have no direct data on long-distance relationships in this regard, day-to-day observations from our work suggest these polycentric structures depend on personal ties embedded not simply in a shared commitment to forest protection, but in business, marriage, friendship and other relationships—ties that both contribute to and result from the existence of polycentric structures. That said, these ties do have a dark side: they may enable the use of private information, such as timing of patrols or the corruptibility of various personnel, in the service of individual harvesting.

In summary, ties between individuals living in different communities can both enhance and erode the success of community forest management.

### (b) Fisheries management in mainland Tanzania

Fisheries have porous boundaries that often do not map on to community boundaries, making community-based management of open-access fisheries difficult. The problem is exacerbated when fisheries are migratory, especially over vast distances; as a result, resource extractors are also frequently migratory, moving across community boundaries to follow preferred fishing populations [81,82]. Moreover, local fisheries are often integrated in regional and international markets, and these market relationships at a distance often affect local behaviour. For example, in Zanzibar fishermen are incentivized to overfish pelagic reef fish to sell to hotels that service international tourists [83], and along the mainland coast people overexploit sardine populations, harvests of which are exported primarily to the Democratic Republic of Congo [84]. Migratory patterns and regional or international market integration provide opportunities to foster long-distance relationships, which can further hinder polycentric NRM by allowing for more leakage, or conversely, help NRM by mitigating conflicts between heterogeneous communities and sharing NRM-relevant information.

In Tanzania, marine resources are largely managed in a participatory co-management scheme, predominantly through CPRUs called Beach Management Units. These institutions were first introduced in Lake Victoria in 2004 by Kenya, Tanzania and Uganda as part of an European Union development plan before being adopted on the coast in 2006 [85,86]. Beach Management Units are associations of fishery stakeholders, including fishers, fish traders, fish processors and boat owners, at a particular coastal landing site [87]. They are formally led by an elected executive committee that works with village and district government officials to protect marine resources via patrols, periodic reef closures and beach cleanings. In the past, Beach Management Units were also able to exclude non-local users via licensing to fish within the administrative district, but since 2019, licenses now allow fishing anywhere within the body of water where the license was issued (e.g. all Tanzanian waters in the Indian Ocean), undermining Beach Management Units' ability to define and exclude users [2]. Fishers commonly come from neighbouring villages or towns to fish for the day and return home, but may come from further away and spend months at ‘camp’ (*dago* fishers).

Increasingly, with the encouragement of the state, NGOs are helping Beach Management Units organize into Locally Managed Marine Areas (referred to locally as Collaborative Fishery Management Areas) to coordinate management. These polycentric systems of governance are led by a coordination committee elected by the constituent Beach Management Unit executive boards. The coordination committee drafts a shared management plan, which the individual Beach Management Units then implement [87]. The Locally Managed Marine Area committee also shares information between the Beach Management Unit executive committees. Taken together, the Locally Managed Marine Areas are the primary means of coordinating management across Beach Management Units—but even in this context, long-distance relationships impact management of fisheries.

*Individuals can use their long-distance relationships to increase private harvests* (figure 2*a*). Similar to the situation with forests on Pemba, long-distance relationships can undercut NRM by helping alters increase catches and avoid enforcement. First, patrolling fishing grounds away from shore requires a boat, which almost every Beach Management Unit and Locally Managed Marine Area lacks, so patrols of open waters are conducted infrequently in collaboration with district fisheries officers. When a patrol is occurring, fishers from neighbouring villages or towns who conduct illegal fishing practices, such as blast fishing, are warned by their long-distance relationships in the local village, such as family, friends, or even paid informants, helping them avoid detection. Second, long-distance relationships may further interfere with patrols themselves. In coordinating patrols, Locally Managed Marine Areas will sometimes have Beach Management Unit executive board members from one village patrol a different village to avoid conflicts-of-interest that often make community-based management difficult [88]; however, if those board members maintain long-distance relationships with the patrolled village, then conflicts may still arise. Third, long-distance relationships can also provide access to illegal fishing gear as illegal gear is usually obtained from outside the village where a fisher resides. In short, when cooperation between individuals has negative impacts on the CPRU or polycentric system, long-distance relationships can foster over-extraction and thus undermine successful NRM.

*Long-distance relationships may mitigate conflict between villages* (figure 2*b*). Managing NRM can be a contentious process, especially when users' abilities, resources or preferences are heterogeneous and certain rules of resource extraction are more favourable to some users [2]. This poses a particular problem when communities vary on dimensions that affect natural resource extraction [22], like, in the case of fisheries, gear used for fishing [21,89]. For example, villages sharing the same fisheries often differ in their gear use, access to markets, availability of alternative incomes and norms, which creates heterogeneity in the net benefits of different rules for extraction, resulting in disagreement and conflict between villages and undermining collective action efforts [89–91]. However, connections between villages, and the fitness interdependence that fosters those connections, can potentially mitigate or resolve conflict resulting from heterogeneity across communities, leading to more successful NRM. For example, in Chwaka Bay on Unguja, conflicts arise between villages that use primarily nets versus baskets, as drag nets interfere with basket traps, but kinship relationships between villages stop these conflicts from escalating to violence [92]

*Long-distance relationships help share information about NRM, including about innovative management practices, especially with the assistance of NGO partners* (figure 2*c*)*.* For example, Beach Management Units are often inconsistently funded by fees they collect in collaboration with the village government, and this lack of funding is a major obstacle to successful management. As a result, some enterprising Beach Management Units have implemented projects to generate revenue, such as building and maintaining beehives to sell honey. Neighbouring Beach Management Units learned of this successful project via long-distance relationships between Beach Management Unit executive committee members, and other Beach Management Units are now exploring whether and how to build their own bee hives.

While long-distance relationships can be an efficient way to spread innovations, innovations will vary in their focus, visibility and distribution of benefits, which may make some innovations more difficult to spread without additional assistance [93,94]. Polycentric governance at higher levels of organization, such as the Locally Managed Marine Area, can help facilitate the spread of information and innovations at lower levels, such as the Beach Management Unit (figure 2*d*). Accordingly, NGO partners often help to facilitate the transmission of innovations by encouraging meetings between Beach Management Units in which they can learn from each other. For example, a Beach Management Unit executive board learned about coral reef restoration practices via an NGO and is beginning to implement those practices. When the Beach Management Unit hosted Locally Managed Marine Area committee elections with the help of the partnering NGO, they invited several representatives to a separate meeting to discuss what the host Beach Management Unit was doing to help coral reefs, which may help spread the practices further [95]. In another example, NGOs in the Western Indian Ocean have been discussing with communities the ecological and economic benefits of periodic reef closures for octopus fisheries [96]. Fishers are often reluctant to implement this practice as it means forgoing some harvest for a season [96,97]. To help fishers adopt periodic closures, when a reef reopens, NGOs bring in representatives from neighbouring Beach Management Units to observe the opening, in the hopes that they will tell others about the value of periodic closures.

## 5. Discussion

In the Anthropocene, managing global commons, such as greenhouse gasses in the atmosphere or fish stocks on the high seas, is challenging because local activities incentivized by global demands, such as deforestation or reef destruction [98], impact the global commons [3,15,39,99]. Attempts to manage these local activities collectively via international pacts or independently by nation-states have largely been unsuccessful [100], in no small part owing to conflict between countries and lags in cultural evolutionary change [3,60]. One solution is to manage natural resources locally within systems of polycentric governance, in which CPRUs are nested under higher levels of governance that reduce the costs of management—for example, by coordinating monitoring and rules across communities [2,18,19]. Successful polycentric governance of natural resources, however, can be difficult to achieve [12], especially as connections between individuals, groups, or communities that span distance and cross community boundaries can bolster or undercut polycentric systems.

Drawing on the evolutionary and development literatures, we provided case studies from Tanzania to illustrate how long-distance relationships can help or hinder NRM. In forests on Pemba and fisheries on the mainland, long-distance relationships are useful for transmitting new ideas for management or even new ways of using natural resources for profit. Individuals are especially likely to adopt transmitted ideas for management when existing solutions are falling short—for example, when outsiders are harvesting a community's protected forest, or when another community has higher profits because of how they are managing their fishery (figure 2*c*). Incursions from outsiders also generate unexpected dynamics, with communities adopting management institutions, or creating their own polycentric institutions (figure 2*b*), to solve collective action problems.

However, the potential for long-distance relationships to undercut local NRM should not be overlooked. In general, social network ties, of which long-distance relationships are just one of many tie types, can enable individuals to monopolize more resources—like if they have consolidated power [101] or have more family locally than do other individuals [102]. In Pemban forests and mainland fisheries, long-distance alters helped individuals to increase their private harvests by, for example, sharing equipment (such as chainsaws and illegal fishing nets) and information (such as the timing and location of patrols). Leakage—or harvesting in a different resource unit to avoid local sanctions—is fostered by long-distance relationships and undercuts the management of large CPRs, including both forests and fisheries [47,63].

Polycentric institutions offer one potential solution to the costs imposed by long-distance relationships and are especially important to successful management of global commons in the Anthropocene, which will require institutions of different scope and scale working together [13,17]. Given the ease with which long-distance relationships can be formed and maintained via online social networks and other technologies, long-distance relationships may offer a powerful tool for building these polycentric systems [11,31]—even more so given the diversity of identities and group allegiances on which ‘community’ can be built when large CPRs are located in cities or are transnational [11]. Just as Ostrom's design principles apply across scales [2], so might the lessons learned from local long-distance relationships, particularly with respect to sharing ideas, creating homegrown management institutions and jump-starting collective action [20,23,103]. Long-distance relationships thus cut both ways: they can undermine collective action around local NRM, but they can bolster it too.

That said, the scale at which ties beyond the community most directly promote and/or undercut collective action in NRM is, we believe, an issue for further debate and analysis. We see suggestive evidence that regional and global long-distance relationships can undermine NRM. For example, long-distance relationships can advance neocolonialism and extractivism perpetuated by the Global North against the Global South [104]; this is sometimes encapsulated in the relationship between international aid organizations and local common-pool resources, with organizations gaining benefits like projects, contracts and substantial employment opportunities by leveraging the challenges encountered in local NRM [105]. On the other hand, long-distance relationships regularly move people [31] and money [106] across borders, and their presence can be a segue to trade [107], peace [108] and large-scale collective action [109]. In short, the relevance of long-distance relationships to local, regional and even international NRM is, in our opinion, worthy of further study.

### (a) Working with long-distance relationships in natural resource management

By being aware of the costs and benefits of long-distance relationships for local NRM, development partners can both anticipate the challenges posed by long-distance relationships for local polycentric systems and use long-distance relationships to support collective action and the adoption of NRM practices. When partners plan an initiative to create or support polycentric systems for local NRM, we recommend they consult with key contacts to assess the impacts of long-distance relationships on the CPR. Leaders and community members may be more forthcoming with development partners about long-distance relationships if a trusting relationship is built first (see [110] for pointers) and may be more willing to report on neighbouring communities than on themselves.

When existing long-distance relationships offer NRM-promoting benefits, partners may consider identifying individuals with strong long-distance relationships and working with them to spread new ideas or management practices [111]—much as an NGO did for fisheries closures on mainland Tanzania. Information sharing is popular, especially among producers (e.g. farmers, fishers, loggers), and can be fostered by organizations [112]. However, the creation and diffusion of common knowledge must involve all, not just the privileged [113–115]—otherwise, as we cautioned above, the privileged may use this information for private gain [110,113,116].

If there are few individuals with long-distance relationships, partners may consider cultivating them by funding field trips to other communities, which can foster connections [103,117,118]. Committed people, such as entrepreneurs, can be especially powerful individuals to send as they may be motivated to promote collective action, even at a cost to themselves [21,119,120]. Partners may consider first building within-community linkages by engaging community members who are eager to be involved, then foster between-community linkages once within-community linkages are strong ([121], cf. [122]). Partners can then encourage communities to set up their own polycentric institutions, as occurred organically in the case of joint forest management in Pemba.

When existing long-distance relationships undercut successful local NRM, partners may consider how to intervene. By using well-known design principles—for example, by providing outside monitors or creating an overarching institution [2,123]—partners may disrupt existing long-distance relationships. Further, when common-pool resources are smaller and can be accessed by fewer potential users, making their boundaries more defensible, partners may consider promoting between-community *competition* rather than between-community connections [3,15,99]—for example, competition between neighbouring bays to best manage lobster stocks [124]. Either way, any successful intervention will need to use the schisms and alliances among individuals at different intervention sites to make collective action work [125].

## 6. Conclusion

The Anthropocene is characterized by increased population sizes, higher mobility and increased demand for resources, all of which are challenging the management of natural resources globally. Humans have a deep history of forging long-distance relationships to maintain resource access, and these long-distance relationships can strain common-pool resources like forests and fisheries even further. However, long-distance relationships often provide resource access because they are interdependent, cooperative connections, and this interdependence can provide the scaffolding for successful local resource management. Rather than fight an uphill battle against a recurrent feature of human social life, development partners may consider ways to use existing long-distance relationships, or to even build new ones, to promote interdependence, coordination and the sharing of ideas. Human social relationships can be an asset, not just a hindrance, to local NRM in the Anthropocene.

## Data Availability

This article has no additional data.
